# Robust Finger Vein ROI Localization Based on Flexible Segmentation

**DOI:** 10.3390/s131114339

**Published:** 2013-10-24

**Authors:** Yu Lu, Shan Juan Xie, Sook Yoon, Jucheng Yang, Dong Sun Park

**Affiliations:** 1 Division of Electronic and Information Engineering, Chonbuk National University, Jeonju 561-756, Korea; E-Mail: luyu0311@gmail.com; 2 Institute of Remote Sensing and Earth Science, Hangzhou Normal University, Hangzhou 311121, China; E-Mail: Shanj_x@hotmail.com; 3 Department of Multimedia Engineering, Mokpo National University, Jeonnam 534-729, Korea; E-Mail: syoon@mokpo.ac.kr; 4 College of Computer Science and Information Engineering, Tianjin University of Science and Technology, Tianjin 300222, China; E-Mail: jcyang@tust.edu.cn; 5 IT Convergence Research Center, Chonbuk National University, Jeonju 561-756, Korea

**Keywords:** finger vein, ROI localization, edge operator, segmentation, orientation correction

## Abstract

Finger veins have been proved to be an effective biometric for personal identification in the recent years. However, finger vein images are easily affected by influences such as image translation, orientation, scale, scattering, finger structure, complicated background, uneven illumination, and collection posture. All these factors may contribute to inaccurate region of interest (ROI) definition, and so degrade the performance of finger vein identification system. To improve this problem, in this paper, we propose a finger vein ROI localization method that has high effectiveness and robustness against the above factors. The proposed method consists of a set of steps to localize ROIs accurately, namely segmentation, orientation correction, and ROI detection. Accurate finger region segmentation and correct calculated orientation can support each other to produce higher accuracy in localizing ROIs. Extensive experiments have been performed on the finger vein image database, MMCBNU_6000, to verify the robustness of the proposed method. The proposed method shows the segmentation accuracy of 100%. Furthermore, the average processing time of the proposed method is 22 ms for an acquired image, which satisfies the criterion of a real-time finger vein identification system.

## 1. Introduction

Recent advances in information technology and the increasing demand for security have led to a rapid development of biometric-based automatic personal identification systems [1]. Traditional biometric characteristics include face, fingerprint, palm-print, iris, retina, gait, hand geometry, *etc.* Biometric-based identification systems using these media have been successfully used in many safety access applications such as customs, airports, and banks. However, no biometric has been proved to be perfectly reliable, robust, secure and cost effective. For example, palm-prints and fingerprints are susceptible to being forged, as they are exposed, being outside the human body. Face recognition is affected by illumination and occlusion. Iris recognition is unfriendly due to the need for direct application of light into the eyes [2]. The tremendous growth in the demand for more user friendly and secured biometrics systems has motivated researchers to explore new biometric features and traits [3].

As a newly emerging biometric technology, finger vein identification has attracted considerable attention in the biometric recognition field. Compared with the other more traditional biometrics, finger vein recognition technology has the benefits of high anti-counterfeiting strength, small imaging devices, low cost, easy collection of images with contactless operation universality, and liveness [4,5]. Furthermore, since the veins are located internally within the living body, the finger vein identification system is less affected by the outer skin surroundings (skin disease, humidity, dirtiness, *etc.*). Hence, finger vein recognition is considered to be one of most promising solutions for personal identification in the future [6].

Over the past decade, finger vein recognition technology has produced remarkable achievements. According to the existing research, finger vein images are easily affected not only by external factors such as scattering [7], uneven illumination [8], and ambient temperature [9], but also by factors appearing in the collection process, e.g., translation, orientation, rotation, scale, finger pressure, and collection posture. Hence, robust ROI localization is crucial for a finger vein identification system. It directly results in whether the interest regions from the same finger vein images have high similarity. To a large extent, the robustness of the ROI localization method determines the performance of the finger vein identification system.

Unfortunately, most of the investigations have focused on finger vein extraction [10–13], finger vein image enhancement [6,7,14,15], novel finger vein feature representation methods [16–20], and multimodal recognition systems that combine the finger veins with other biometrics [9,21,22]. Little research has been devoted to finger interest region exploitation. Furthermore, most of the currently utilized finger vein ROI localization methods are, in practice, sensitive to finger position variation [6]. To solve the problem, Yang *et al.* proposed a finger vein ROI localization and vein ridge enhancement method [6]. The finger structure and optic knowledge are combined to localize the ROI. This works well for most finger vein images, but the method fails for some images since the influence of image orientation is ignored, although the influence of image orientation has been alleviated with a considerable imaging device.

In this paper, we propose and detail a robust finger vein ROI localization method. Two extended edge operators are employed first for coarse finger region segmentation. This has been verified to result in faster speed than those using the masks [22]. For the images in the abnormal case, elaborate binarization is proposed to remove the false background caused by the influences from uneven illumination, scattering, and improperly collection. To do this, secondly, the extended edge operators on the enlarged image are processed to solve the influence from improper collection, followed by a single linkage clustering algorithm to delete wrong middle edge points, resulting from the influence of uneven illumination and scattering. Afterwards, the calculated orientation angle is employed for orientation correction. The removal of false backgrounds and estimated corrected orientation angle can be guides for each other to get a more accurate finger region. Finally, an extended ROI detection method is employed based on searching the reference line in the second knuckle of the finger. To alleviate the influences from the uneven illumination and variation of finger width, the reference line is derived from the vertical projection of block image truncated from the segmented or orientation corrected image. Experimental results on our established database (MMCBNU_6000), including 6,000 images from 100 subjects, have demonstrated that the proposed method is highly feasible and robust for the influence factors from translation, orientation, scale, finger gesture, scattering, and illumination, which shows the segmentation accuracy of 100%.

The remainder of this paper is organized as follows: Section 2 reviews state-of-the-art technologies concerning the ROI localization method. A detailed description of our robust ROI localization for finger vein recognition is given in Section 3. Section 4 presents a portable finger vein imaging device and provides extensive experimental results that verify the proposed method. The conclusion of this paper and ideas for further exploration are summarized in Section 5.

## 2. Related Works

ROI localization is an indispensable process for a finger vein identification system to explore the effective region from the acquired image. It largely determines the performance of the identification system. ROI localization is usually composed of finger region segmentation, image orientation correction, and ROI detection. Since ROI localization is an indispensable process for a finger vein identification system, most of the researches have briefly reported their ROI localization methods. Here, we briefly review the ROI localization methods.

Finger region segmentation aims to segment the finger region from an acquired image with a complicated background. For finger region segmentation, there are basically three kinds of methods: the thresholding method, the finger edge detection based method, and their combination. In [17], the captured image was binarized using a threshold value that has been determined by Otsu's method. Then the center of the object was considered as the finger region. Kumar *et al.* [9] subjected the acquired finger vein image to binarization using a fixed threshold value of 230, to coarsely localize the finger shape. Afterwards, the isolated and loosely connected regions in the binarized image were eliminated, using the Sobel edge detector and area thresholding. Song *et al.* [12] detected the outlines of the finger by thresholding the Laplacian of the image, and then found the boundaries closest to the horizontal center line. The finger edge detection based method aims to find the upper and lower finger edges, using different edge operators. The Sobel operator was used for detecting the edges of a finger in [19]. The width of the finger region was then obtained based on the maximum and minimum abscissa values of the finger profile, while the height of the finger region could be similarly detected. In [23], a Canny operator with locally adaptive threshold was applied to get the single pixel edge of the finger. Xie *et al.* proposed to extract the binary edge image using the Hough transform [15]. Unfortunately, these methods are not reliable and robust for images with complicated backgrounds, influences from scattering and uneven illumination.

The purpose of image orientation correction is to rotate the images to the same base angle (vertical or horizontal orientations). Less detailed works have been introduced to solve image orientation correction. Although the image orientation can be alleviated by designing a good imaging device with guidance, the acquired images are more or less affected by the orientation, due to different shapes of different fingers, especially for images acquired from thin fingers. In [23], the midline obtained from the midpoints of the upper and lower finger edges was utilized to horizontally adjust the finger. According to two peaks corresponding to two horizontal finger contour lines after a Hough transform operation, Xie *et al.* corrected the orientation of the image when the two detected peaks satisfied the set condition [15]. In this way, the performance of image orientation correction is highly dependent on the accuracy of the detected finger edges. However, factors from image and camera noise always degrade the performance of finger edges detection, especially the influence from segmentation errors.

ROI detection focuses on the extraction of the region of interest from the orientation corrected image. There are basically three kinds of methods to detect the ROI. The first method is directly considering the segmented image as the ROI [9,24]. This method has good performance under the assumption that the influences of translation, orientation, scale, *etc.*, have been alleviated by the imaging device. The second method usually captures a fixed rectangle region from the segmented image or orientation corrected image. Basically, the rectangle region is less than the captured image. Xie *et al.* determined the ROI with a rectangle region from the center of the rotated image [15]. In [17], the center of the finger region was extracted from the segmented image to be the ROI. Wu *et al.* set a fixed region to extract the ROI [18]. However, the ROIs detected using these two methods are easily affected by translation and scale, notwithstanding the good design of the imaging device. The third method [6,23,25,26] extracts the ROI based on a reference line in the image. Since the bone in the finger joint is articular cartilage, and it can be easily penetrated by infrared light, the joint part in the image is brighter than that of other parts. Hence, after the horizontal image projection of the orientation corrected image, the peaks of the projection curve correspond to the approximate position of the joints. These peaks can be utilized to be the reference points for ROI detection. However, the methods [23,25,26] are easily affected by uneven illumination and intensity variation resulting from the finger structure. To solve the problem, a sub-region that is less than the finger vein region is located for the vertical projection of pixel values in [6].

The existing methods are not perfectly reliable and robust for the influences from translation, orientation, scale, collection of image, uneven illumination, background of imaging device, *etc.* Hence, we are encouraged to explore a robust ROI localization method for finger vein imagery.

## 3. Robust Finger Vein ROI Localization

**Figure 1.**
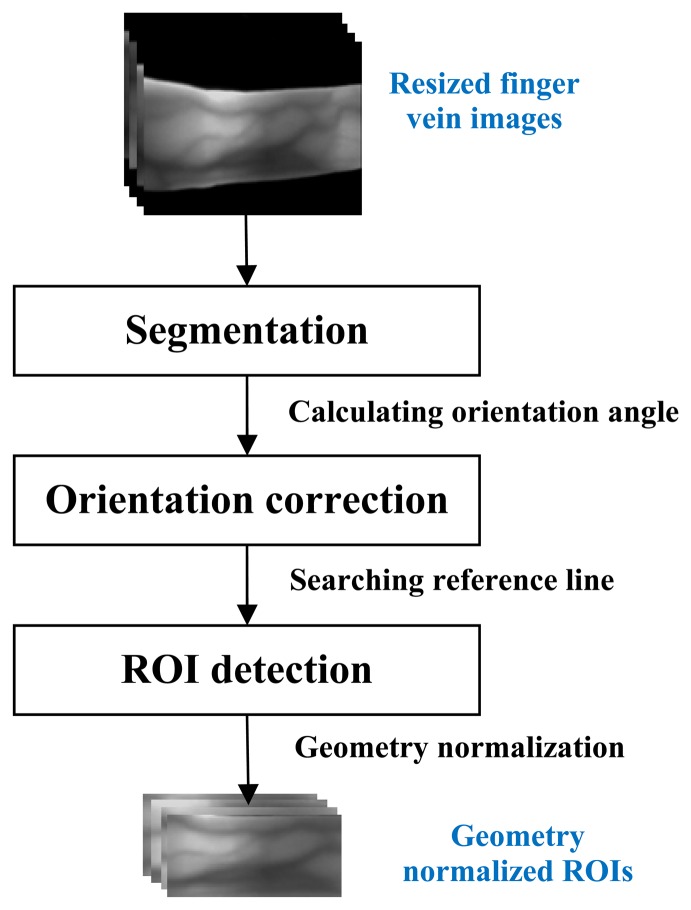
Block diagram illustration of the proposed method.

An effective and robust finger vein ROI localization method is essential for a finger vein identification system. In this section, we detail the proposed method, which consists of segmentation, orientation correction, and ROI determination, and which is illustrated in Figure 1. In order to reduce the processing time, the acquired image with 480 × 640 pixels size is resized to 120 × 160 primarily with “bicubic” interpolation. The details of each step are described as follows.

### 3.1. Segmentation

**Figure 2.**
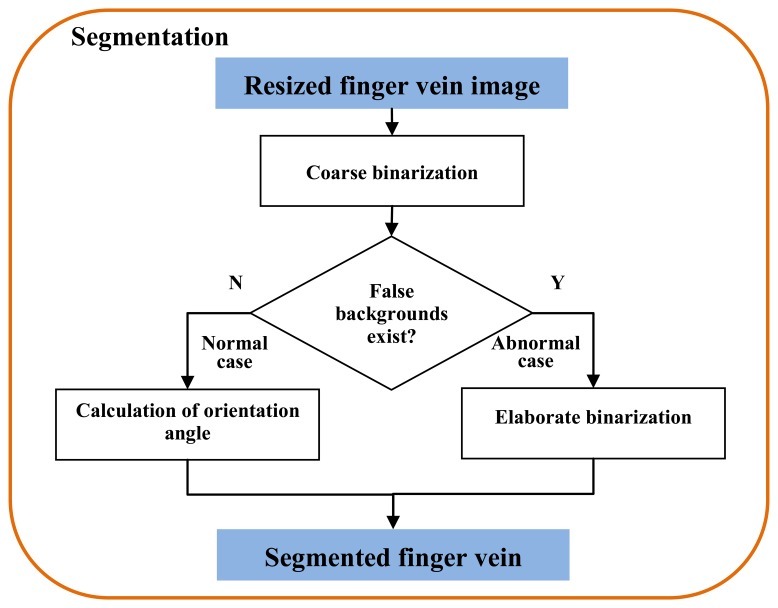
Block diagram of segmentation.

Owing to the complicated background of the captured images, the acquired finger vein images are divided into two cases: normal case and abnormal case. This is decided according to whether false backgrounds exist in the coarse binarized image. The images without false backgrounds appearing in their coarse binarized images are considered as the normal case. Otherwise, it is identified as the abnormal case. Figure 2 shows the block diagram of the segmentation process in this paper. For a coarse binarized image without false background, the orientation angle is computed for image orientation correction. For those with false background, elaborate binarization is necessary for accurate finger region segmentation.

#### 3.1.1. Coarse Binarization

**Figure 3.**
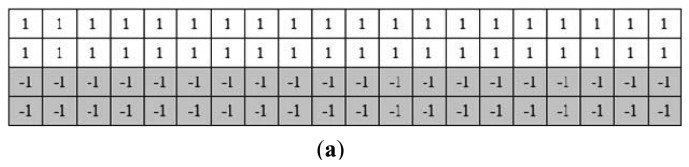

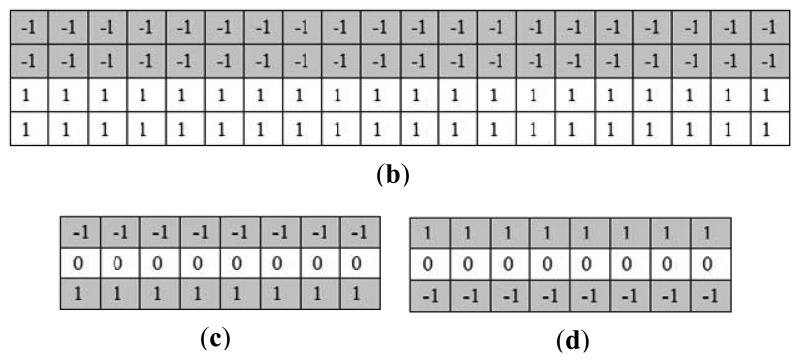
Masks for localizing the finger region from the resized acquired image. (**a**), (**b**) masks used in the method [22]; (**c**), (**d**) the proposed edge operators.

**Figure 4.**
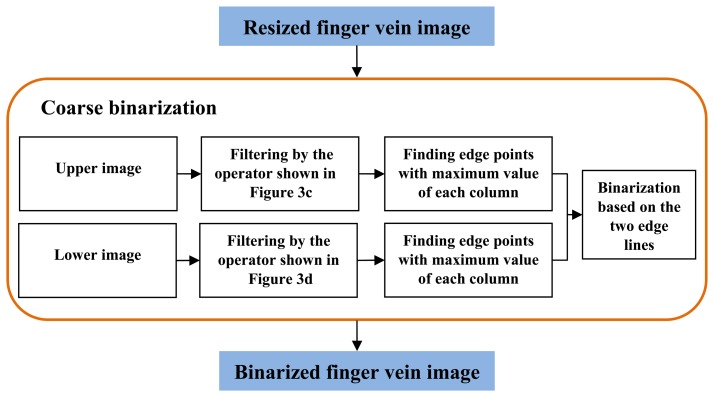
Block diagram of coarse binarization.

For the finger vein images in the normal case, the finger region is segmented using the binarized image. In the normal case, since the finger region is brighter than the background region, it is easy to localize the finger region from the captured image. In [22], a finger vein image is horizontally divided into upper part and lower part of the same size. Then, two masks (Figure 3a,b) is utilized for finger region binarization. The essential idea of this method is to detect two edge lines from the upper and lower images. With the mask operation, the contour of the finger can be localized, by detecting the maximum values for each column from the filtered image. The region between the detected upper contour and lower contour is then binarized to 1, to be the foreground. On the contrary, the pixels outside the finger region are set to 0, to be the background. Inspired by it, an extended operator shown in Figure 3c,d that extends the Prewitt operator is proposed to segment the finger region. The process of coarse binarization is described in Figure 4.

**Figure 5.**
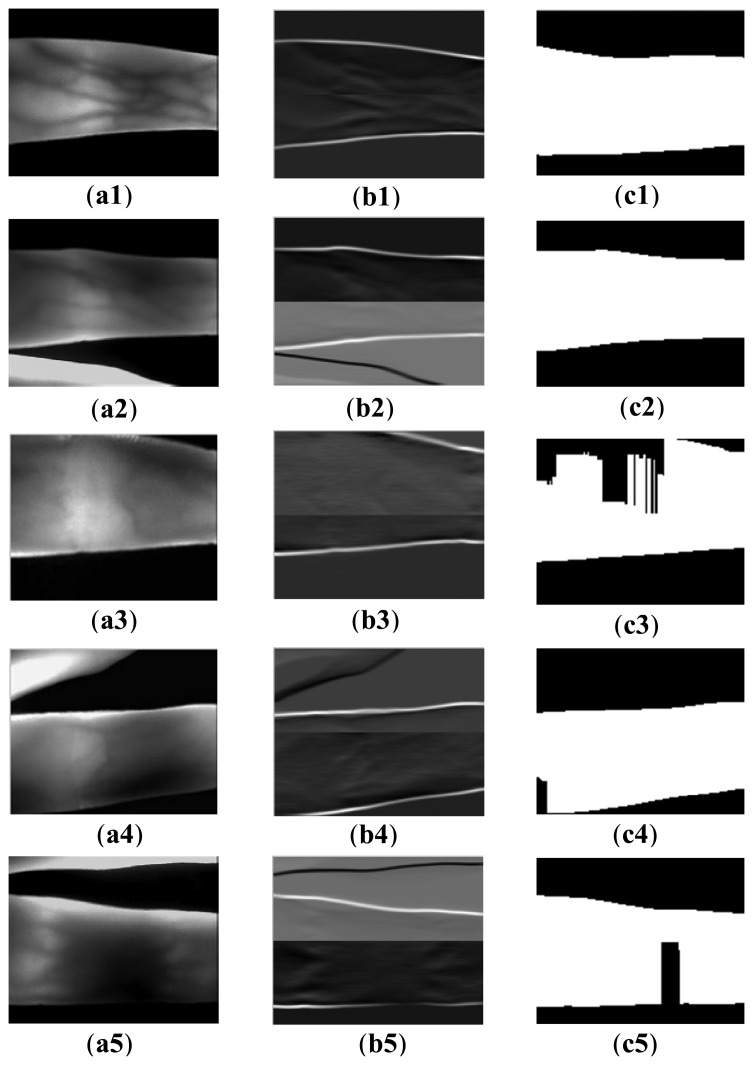
Coarse binarization results using the proposed edge operators. (**a1**–**a5**) resized acquired image samples; (**b1**–**b5**) the filtered images using the proposed operator; and (**c1**–**c5**) binarized finger regions (segmentation results).

Figure 5 displays the binarization process of the finger region using the proposed edge operators. A given image (e.g., Figure 5a1) is divided into two sub-images, upper one and lower one. The masks of Figure 3c,d is then applied to upper one and lower one respectively, according to the direction from the center line to boundary lines of original image. Because of this directionality of each filter mask, the change from brighter one to darker one make a bright line and the change from darker one to brighter one make a dark line. The upper and lower filtered images are then normalized to 0–255, and combined together to form an integral filtered image (e.g., Figure 5b2). For the filtered images, Figure 5b1,b2 clearly shows that two complete edges appear in the upper and lower part of the filtered images. Even for the image (Figure 5a2) with the useless finger part, and influence from uneven illumination affected by absorption of the useless finger, the proposed edge operators can also segment the finger region well. However, the proposed operator is not robust for the cases shown in Figure 5a3–a5. Since the upper finger contour is not completely shown in the captured image (Figure 5a3), there is a broken finger edge in the upper part of the filtered image. The same case with false background appears in the left lower part of Figure 5a4. Although a very small finger region disappears in this kind of acquired image, it can also be recognized according to our experimental result, with the assumption of accurate finger segmentation result. The false background that exists in Figure 5c5 is attributed to scattering [7] and uneven illumination [8]. The scattering effect can greatly reduce the contrast between the venous and non-venous region [27]. In addition, the useless finger region shares the infrared light, which results in the lower finger part absorbing less infrared light than the upper part. Hence, a false background exists in its binarized image.

#### 3.1.2. Elaborate Binarization

**Figure 6.**
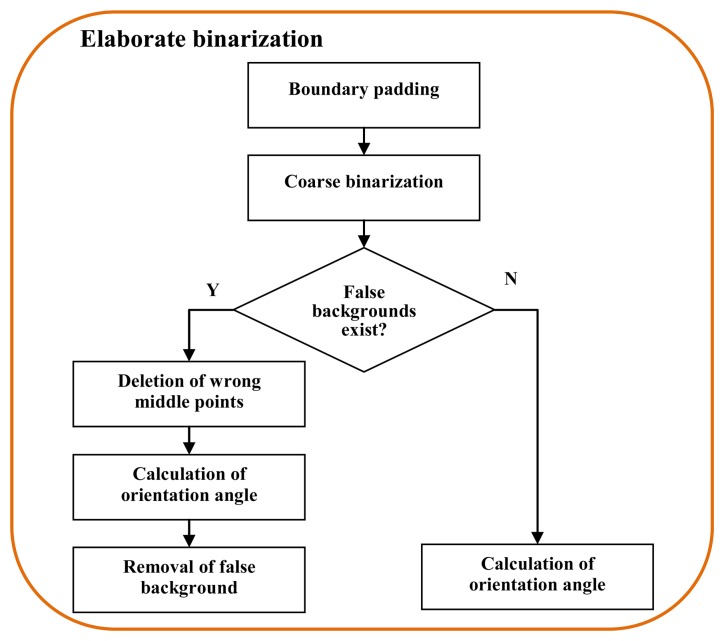
Block diagram illustration of elaborate binarization.

For the images in the normal case, the correct binarized finger region can guide the finger orientation correction. Unfortunately, false backgrounds exist in some binarized images because the acquired images correspond to the abnormal case. Hence, additional processes are necessary to obtain an accurate binarization result. To do this, elaborate binarization is designed for the correct segmentation. Figure 6 shows the block diagram of elaborate binarization for the images with abnormal background. Each step is reported in detail, as follows.

##### Boundary Padding and Coarse Binarization

It can be ascertained that the proposed operator can correctly binarize the finger region whose edges are clear and complete in the filtered image. Inspired by this, an ideal method to segment the images with false background in the coarse binarization results (Figure 5a3,a4) is to pad the boundary for them. This method has the assumption that the image boundaries can be directly considered as the finger edges, because only a small finger region disappears in these images.

To this end, it is firstly verified as to whether the false background is caused by the disappeared edge in the upper or the lower part. Assume *I*(*x*, *y*) ∈ *R^m^*^×^*^n^* is a resized finger vein image. *up* = {*up*_1_, *up*_2_, ⋯, *up_n_*} and *low* = {*low*_1_, *low*_2_, ⋯, *low_n_*} are the Y-axis values from the detected upper and lower edge points, respectively. Each group of edge points is divided into two parts with the same size, *up* =*up^I^* ∪ *up^II^* , and *low* =*low^I^* ∪ *low^II^* . Then the variances of each part from the upper and lower edge points are calculated, as follows:

(1) $$\text{Var}\_{up}^{I}=\mathrm{var}({up}^{I}),{up}^{I}=\{{up}_{1},{up}_{2},\cdots ,{up}_{n/2}\}.$$

(2) $$\text{Var}\_{up}^{II}=\mathrm{var}({up}^{II}),{up}^{II}=\{{up}_{n/2+1},{up}_{n/2+2},\cdots ,{up}_{n}\}.$$

(3) $$\text{Var}\_{\mathrm{low}}^{I}=\mathrm{var}({\mathrm{low}}^{I}),{\mathrm{low}}^{I}=\{{\mathrm{low}}_{1},{\mathrm{low}}_{2},\cdots ,{\mathrm{low}}_{n/2}\}.$$

(4) $$\text{Var}\_{\text{low}}^{II}=\mathrm{var}({\text{low}}^{II}),{\text{low}}^{II}=\{{\text{low}}_{n/2+1},{\text{low}}_{n/2+2},\cdots ,{\text{low}}_{n}\}.$$

**Figure 7.**
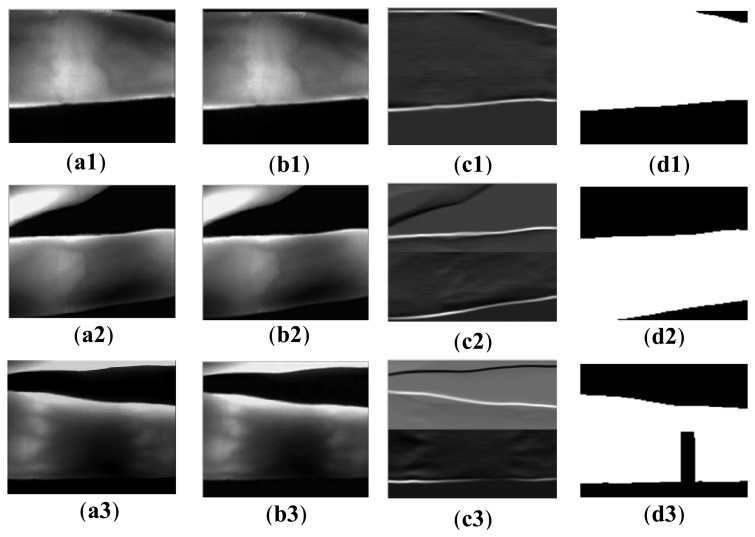
Re-binarization of the finger region using the proposed masks: (**a1–a3**) resized acquired image samples; (**b1–b3**) padding images; (**c1–c3**) the filtered images, and (**d1–d3**) final binarized images.

A threshold *Thr* is set to verify whether the upper part or lower part has the disappeared edge. The disappeared edge is in the upper part if *Var* _*up^I^* >*Thr* ‖*Var* _*up^II^* >*Thr* , while the disappeared edge is in the lower part when *Var* _*low^I^* >*Thr* ‖*Var* _*low^II^* >*Thr*. With the position in which the disappeared edge is located, a new finger vein image *I_New_*(*x*, *y*) with resolution of (*m* + *t*)×*n* is built, by padding *I* (*x*, *y*) . The padding region *t*×*n* at the top of Figure 7a1 has the same intensity value 0 as the background. Similarly, a region *t*×*n* with intensity value 0 is padded at the bottom of image *I* (*x*, *y*), for the second case (Figure 7b2,b3). Here, the threshold *Thr* = 38 is an empirical value, which is determined according to the experimental results. *t* is determined as 3 to reduce the processing time.

Figure 7 shows the key steps of the re-binarization of the finger region using the proposed edge operators. As shown in Figure 7b1–b3, since only three rows are padded, the padding regions are not obvious. Compared with the filtered images shown in Figure 5c3,c4, the detected edges are unbroken, as shown in Figure 7c1,c2. With the integrated edge, accurate finger regions (Figure 7d1,d2) are segmented from the resized acquired images. Afterward, the final binarized image is obtained, by discarding the part of *t*×*n* in the top or bottom of the re-binarized image. For these images, the accurate segmented finger region can guide the finger orientation correction.

Unfortunately, the process of re-binarization is also not robust for the kinds of images shown in Figure 7a3. The reason is that the broken edge results from scattering and illumination, other than the disappeared finger part. The same binarization errors occur in the final binarized image (Figure 7d3). To solve this problem, the calculated orientation angle is employed to guide the removal of the false background.

##### Deletion of Wrong Middle Points

**Figure 8.**
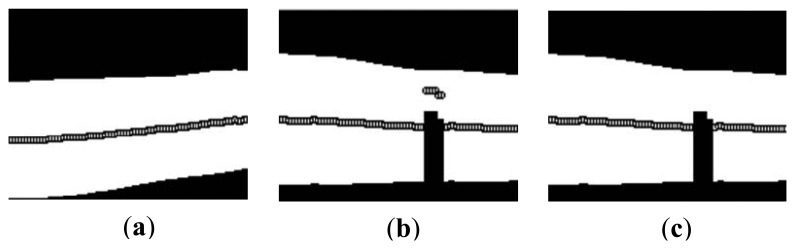
Display of the calculated middle points of the finger region in: (**a**) correct binarized finger region; (**b**) binarized finger region with binarization errors; and (**c**) binarized finger region after deletion of wrong middle points.

In this paper, middle points obtained from the detected upper and lower edge points are employed, to guide the image orientation correction [23]. For the image with correct segmentation (Figure 8a), middle points obtained from the upper and lower edge points can be employed to estimate the orientation angle of the acquired image. However, the segmentation errors appeared in Figure 8b will degrade the accuracy of calculating the orientation angle, which results in inaccurate orientation correction. Hence, it is necessary to delete the wrong middle points located in the segmentation errors. Assuming *I_B_*(*x*, *y*) is the binarized image, we obtain the Y-axis values of the middle points from the finger region:

(5) $$y_{i}=\frac{{up}_{i}+{\text{low}}_{i}}{2},i=1,2,\ldots ,n,$$

where *up_i_* and *low_i_* are the upper and lower detected edge point on the Y-axis, respectively. The middle points (*x_i_*, *y_i_*), *i* =1,2, … , *n* are marked by ‘o’ as shown in Figure 8.

A single linkage clustering method [28] is applied to delete the inaccurate middle points to estimate the facial orientation in [29]. Furthermore, an iterative strategy is developed to prevent random choice of the link distance to get the final estimated orientation. The method can be extended to detect arbitrary objects with mirror symmetry or near symmetry property. Inspired by this, we apply the single linkage clustering method in this paper, to delete the wrong middle points. Since the operator of re-binarization has largely decreased the number of wrong middle points, the iterative strategy proposed in [29] is redundant for the orientation estimation in this paper. By using the single linkage clustering algorithm, the wrong middle points ( $x_{i}^{w}$, $y_{i}^{w}$), *i* =1, 2, … , *p* (*p* is the number of incorrect middle points) in the false background (Figure 8b) can be deleted. Figure 8c shows the binarized finger region with accurate middle points.

##### Calculation of Orientation Angle

With the accurate middle points, the orientation angle can be calculated using least-squares estimation (LSE). Assuming the line function of the finger is described by *y* = *kx* + *b* , then the parameters can be directly derived according to least-squares estimation (LSE):

(6) $$k=\overset{n}{\underset{i=1}{\text{∑}}}\left(x_{i}-\bar{x}\right)(y_{i}-\bar{y})/\overset{n}{\underset{i=1}{\text{∑}}}{\left(x_{i}-\bar{x}\right)}^{2},$$

(7) $$b=\bar{y}-k\bar{x},$$

(8) $$\begin{array}{cc} \bar{x}=\frac{1}{n}\overset{n}{\underset{i=1}{\text{∑}}}x_{i}, & \bar{y}=\frac{1}{n}\overset{n}{\underset{i=1}{\text{∑}}}y_{i}, \end{array}$$

where *x_i_* = 1, 2, …, *n*, *i* =1,2,…, *n*,. Hence, the corrected orientation angle *θ* between X-axis and the estimated line can be computed as follows:

(9) $$\theta =\{\begin{array}{cc} -\arctan(k), & k<0,\\ \arctan(k), & k\geq 0. \end{array}$$

##### Removal of False Backgrounds

**Figure 9.**
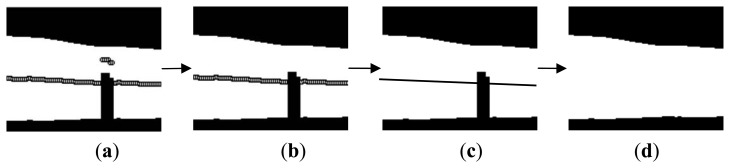
Processes of removing the false background. (**a**) binarized image with all middle points; (**b**) binarized image with correct middle points; (**c**) segmented image with the estimated line calculated from the correct middle points; and (**d**) integrated finger region after removing the false background.

The estimated orientation corrected angle can not only be applied for finger vein image orientation correction, but also employed in filling the broken region for those images with false backgrounds. Assume *θ* is the estimated finger orientation. With *θ* and the values of X-axis coming from the wrong middle points ( $x_{i}^{w}$, $y_{i}^{w}$), *i*=1, 2, …, *p* , we can calculate their correct values of Y-axis $y_{i}^{c}$, *i* = 1, 2, …, *p* using the line function *y* = *kx* + *b*. Furthermore, the corresponding values of Y-axis of the disappeared edge points can also be computed. Thus, the accurate estimated middle points ( $x_{i}^{c}$, $y_{i}^{c}$), *i* = 1, 2, …, *p* can be directly guided to remove the false background. Figure 9 shows the processes to remove the false background. With this operation, the proposed method is robust for segmenting all of the captured images.

### 3.2. Orientation Correction

In this part, the segmented images are rotated according to the estimated orientation angle. For the images with false backgrounds (e.g., Figure 9a), the orientation corrected angle *θ* can be employed to correct the orientations of the correct segmented images. Since there is no false background in the binarized images obtained from the acquired images with correct background, all the middle points are utilized to compute *θ* using the LSE method. Hence, with the orientation corrected angle *θ*, the segmented images can be rotated using “bicubic” interpolation. In this paper, the acquired image will be orientation corrected with the condition that the absolute value of *θ* is larger than 1 degree.

### 3.3. ROI Detection

Even though images are acquired from a finger of a person, they may be transition, scale, or rotation-variant issues caused by the acquisition environment such as the user's actions, so it is not guaranteed that the same image can be always obtained from the same object. These variations always cause high inner-class-distance of two images from one individual, and so degrade the matching performance, even for the accurate segmented images. Hence, ROI detection aims to explore the interest regions from the same individual's processed images and make the inner-class-distance as small as possible. An ideal method for ROI detection is to find the reference line for each image [6,23,25,26]. As described in Section 2, the method applied in [23,25,26] is easily affected by the uneven illumination and intensity variation resulting from the finger structure. Hence, the method used in [6] to detect ROI is extended and then employed in this paper. It should be stressed that the ROI detection in this paper may be performed on the correct segmented image or orientation corrected image, rather than the segmented image in [6].

**Figure 10.**
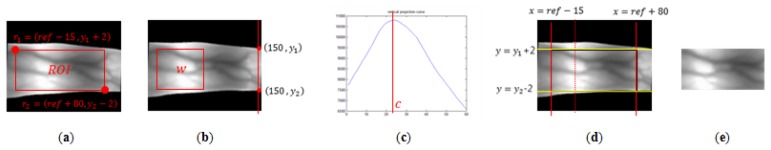
Processes of finger vein ROI detection, (**a**) processed image with *w*; (**b**) curve of truncated image vertical projection; (**c**) ROI with reference line (red); (**d**) localized ROI; and (**e**) geometry normalized ROI.

Furthermore, different from the method based on searching the reference line in the first knuckle [6], in this paper, the ROI of finger vein image is defined as a fixed region, based on the reference line in the second knuckle. This is determined owing to the excogitation of our imaging device. Since near infrared light can easily penetrate the articular cartilage [23], the gray values of the pixels in knuckles are larger than other parts, which results in larger accumulated value of the parts in the knuckles than other regions. Hence, with vertical projection of the image, the column with accumulated value that has maximum value can be the reference line for ROI detection. As shown in Figure 10a, ROI is defined as a rectangle determined by two points, *r*_1_ = (*ref* −15, *y*_1_ + 2) and *r*_2_ = (*ref* +80, *y*_2_ − 2). All constant parameters used for ROI detection are selected through experiments. The procedures for ROI detection are described as follows:

(1) In order to determine two values, *y*_1_ and *y*_2_ , two edge pixels, (*x*_1_, *y*_1_) and (*x*_1_, *y*_2_) are used, where *y*_1_ and *y*_2_ are two ends of Y-axis on the line of *x* = *x*_1_ in the finger vein region. Since the smallest widths from different fingers are almost located in the right terminal of the images, two edge pixels are selected as (150, *y*_1_) and (150, *y*_2_) , as shown in Figure 10b.
(2) The value, *ref* , can be determined by using the position of the second knuckle of a finger. Since second knuckles commonly appear on the left side of finger vein images, a truncated block image *w* obtained from the left part of the image is used to decide the location and reduce processing time. *w* of *m*_1_ × *n*_1_ is determined by two pixels, (20, *y*_1_) and (80, *y*_2_). A block image *w* is then projected in the vertical direction. Figure 10c shows the curve of image vertical projection:
  (10) $$V_{j}=\overset{m_{1}}{\underset{i=1}{\text{∑}}}w(i,j),\;j=1,2,\ldots ,n_{1}.$$
(3) The column with the maximum projected value in the curve, *c* , is chosen to get the reference line, *ref* = *c* + 20, for ROI detection. In Figure 10d, the reference line is displayed with a red dotted line:
  (11) $$c=\underset{j}{arg max}(V_{j}),\;j=1,2,\ldots ,n_{1}.$$
(4) Since the lengths of different fingers vary, the positions of the detected reference lines are different. To extract a ROI as large as possible, the width of the ROI is defined as the region from *ref* −15 to *ref* + 80 , displayed in red solid lines in Figure 10d. Hence, the length of the extracted ROI is 95. Owing to different shapes from different fingers, the height of the ROI is based on *y*_1_ + 2 and *y*_2_ − 2 displayed in yellow solid lines in Figure 10d. In this way, the detected ROI contains the effective foreground (finger region), as much as possible.
(5) Since the height of the ROI varies with different fingers, geometry normalization is necessary to eliminate the geometrical variations. In this paper, all localized ROIs are normalized to 60 × 128 pixels with ‘bicubic’ interpolation, like Figure 10e.

In practical scenario, the collection positions of each finger for several attempts are variant, even there is an elegant design of guidance in a imaging device. Hence, the collected images are more or less affected by the image translation. The influence from image translation can degrade the performance of a finger vein recognition system. Furthermore, due to different individuals have different finger lengths, the values of *c* are located in a wide region. These factors results in the smaller region of ROI (Figure 10e), compared with the original image (Figure 10a). Although the image size of ROI is reduced, the difference between two finger vein images that are from one finger is also reduced.

## 4. Experimental Results and Discussion

In order to verify the feasibility and robustness of the proposed method, extensive experiments are performed on our established database which is named MMCBNU_6000. The images are all captured using a lab-made portable imaging device.

### 4.1. Finger Vein Imaging Device and Dataset

**Figure 11.**
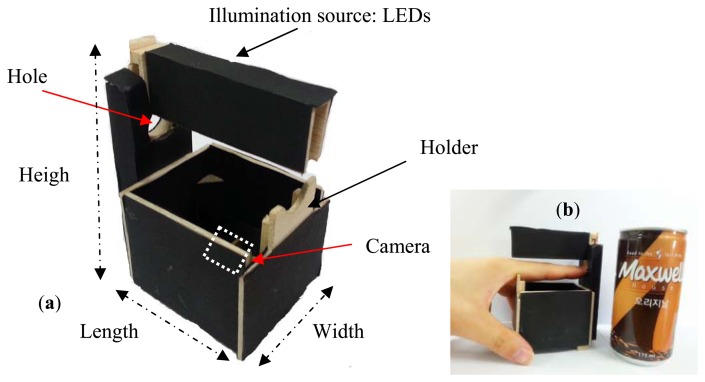
Lab-made Imaging device and example for image collection. (**a**) the portable finger vein imaging device designed by our lab; and (**b**) an example of image collection for the left forefinger.

As shown in Figure 11a, the lab-made imaging device is composed of a camera with an infrared light passing filter and an array of infrared LEDs of 850 nm wavelength. For the sake of improving the convenience for image collection, a holder is added on the back of the device, and a hole is punched that is larger than the thickness of the top of an adult's finger. In addition, a USB interface is applied for power supply to allow the portability. As shown in Figure 11b, the size of our device is 6.8 × 5.4 × 10.1 (length × width × height: cm), which is about the same height as a can of coffee.

MMCBNU_6000 consists of finger vein images captured from 100 volunteers, who are students and professors in CBNU from Asia, Europe, Africa, and America, coming from 20 different countries. The ages of volunteers are from 16 to 72 years old. Statistical information of the nationality, age, gender, and blood type of each volunteer is available for deep analysis on the finger vein image. Since the length of the thumb and the little finger is too short, compared with other three fingers, each subject was asked in the capturing process to provide images from his or her index finger, middle finger, and ring finger of both hands in a standard office environment (rather than a darkroom). The collection for each of the six fingers is repeated 10 times to obtain 10 finger vein images. For each image collection, the subject was asked to input his or her finger optionally. Our finger vein database is composed of 6,000 images. Each image is stored in “bmp” format at 480 × 640 pixels size.

**Figure 12.**
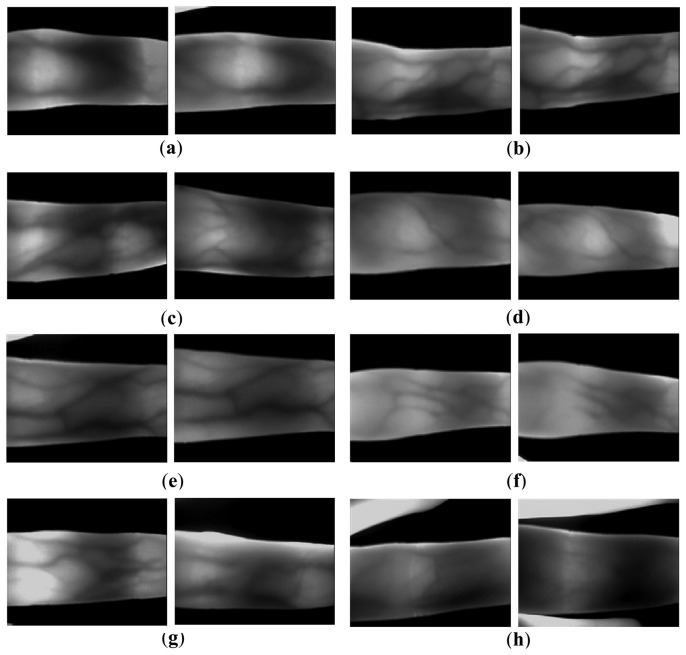
Influences from different factors on the finger vein images. (**a**) translation; (**b**) orientation; (**c**) scattering; (**d**) scale; (**e**) rotation; (**f**) finger pressure; (**g**) uneven illumination; and (**h**) collection posture.

Figure 12 shows the influences of different kinds of factors on finger vein images. All the influences enhance the difficulty of correct ROI localization. Translation, orientation, scale, and rotation, as shown in Figure 12a,b,d,e, may result in wrong regions of interest (ROI), which will increase the probability of erroneous matching. The quality of finger vein images (Figure 12c) is always poor because the scattering effects can greatly reduce the contrast between the venous and non-venous regions [27]. For the effect from pressure shown in Figure 12f, the inaccurate ROI may be obtained even though there is a correct ROI detection. The factor of uneven illumination displayed in Figure 12g will mistake the vertical projection. Moreover, since the finger beside the captured finger shares the IR light, the IR light for the captured finger is not enough. In this way, the vein image from the captured finger is shown blacker than those in the normal case, as shown in Figure 12h. This factor always affects the accuracy of finger region segmentation.

### 4.2. Comparison of Segmentation Performance

In this part, the existing segmentation methods, Otsu's thresholding [17], mask based method [22] and re-binarization using the proposed edge operators, are implemented for comparison.

**Figure 13.**
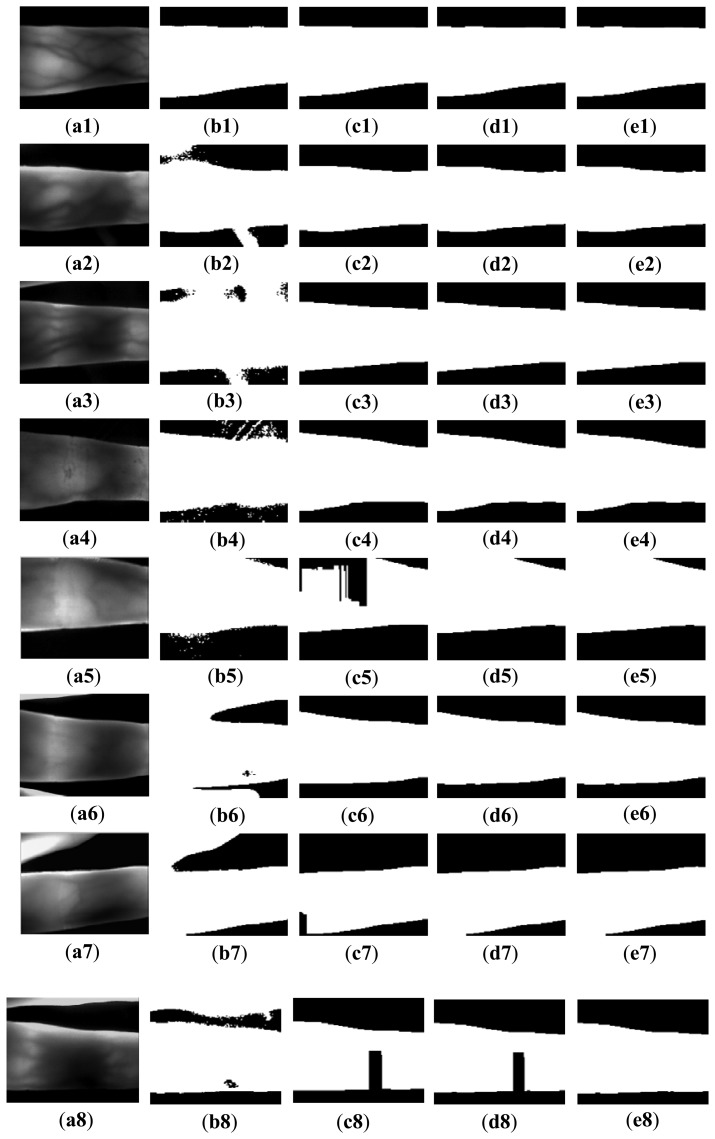
Segmentation results using different methods. (**a1**–**a8**) acquired images, (**b1**–**b8**) Otsu's thresholding method used in [17], **(c1**–**c8**) mask based method used in [22], (**d1**–**d8**) re-binarization using the proposed edge operators, and (**e1**–**e8**) the proposed method.

Figure 13 shows the segmentation results using the above methods and the proposed method. Figure 13a1 is a normal image, with good image background and image quality. Owing to the uneven illumination, the left upper finger region in the image Figure 13a2 is brighter than other regions. There is some parasitic light in Figure 13a2,a3. The image in Figure 13a4 is acquired from a volunteer whose age is 72 years old. The factors causing the complicated background from Figure 13a5 to Figure 13a8 result from image collection for the index and ring fingers, improper collection posture, and scattering. It is ascertained that Otsu's thresholding method and the method using edge mask are not robust for segmentation. For Otsu's thresholding method, some segmentation errors appear in the outputs, due to the influences from illumination, parasitic light, and useless finger parts. The mask based method [22] can solve these errors. However, the method using the mask is invalid for the influence from scattering and incomplete finger.

Hence, these two methods are not robust for some variations from the external and internal factors. As shown from Figure 13e1 to Figure 13e8, the proposed method can correctly segment the acquired images with the influences from illumination, scattering, parasitic light, and useless background. Compared with the results (Figure 13e1–e8) using the mask based method [22], the proposed method has higher robustness than the masks displayed in Figure 3a,b. With the accurate edge information, an accurate orientation correction angle can be estimated to guide the image orientation correction.

In order to compare the robustness of the proposed method with other methods, the segmentation accuracies obtained using different segmentation methods are computed. The output image without false background is considered as a correct segmentation, as the images shown in Figure 13e1–e8. Otherwise, the output with false background (e.g., Figure 13b2–b8,c5) is identified as a false segmentation. The segmentation accuracy can be computed as follows:

(12) $$\text{Accuracy}=\frac{\text{number of correct segmented image}}{\text{total images in database}}.$$

As shown in Table 1, the robustness of the proposed method is confirmed from the segmentation accuracy of 100%, achieved using MMCBNU_6000. Otsu's thresholding method is highly sensitive to the influences from illumination and background, which brings poor segmentation result. The mask based method also has high segmentation accuracy, duo to the good image background of the images in our database.

Table 2 depicts comparison of the processing time using different segmentation methods. All the experiments are performed using MATLAB (R2010a) on a computer with Intel Core i3-2120 and 4 GB RAM. From the results as shown in Table 2, the proposed operator has less processing time than the mask used in [22], which shows the average processing time of 7.7 ms for the coarse binarization. In addition, the processing time of the proposed method includes the time for image segmentation, image orientation correction, and ROI detection. The total processing time using the proposed method to localize the ROI from an acquired image is 22 ms, which satisfies the criterion of a real-time identification system.

### 4.3. Comparison of Orientation Correction Performance

**Figure 14.**
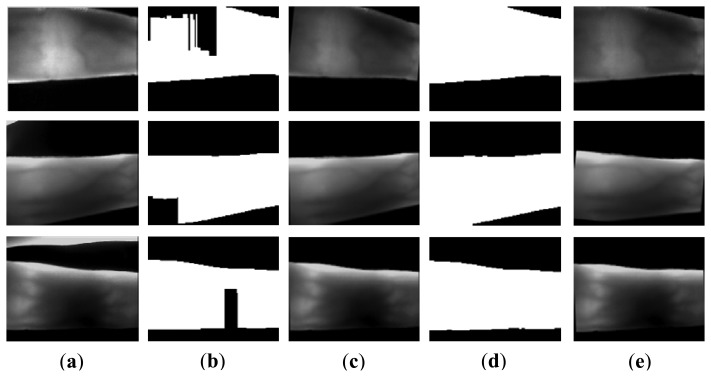
Orientation correction results using different methods. (**a**) acquired images; (**b**) segmented images using the mask based method proposed in [22]; (**c**) orientation corrected images according to the segmented result of (**b**); (**d**) segmented images using the proposed method; and (**e**) orientation corrected images according to the segmented result of (**d**). For the rotated image in the second row of (**e**), better vision effect can be obtained when you focus on the lower finger contour.

In this section, we compare the orientation correction results with, and without elaborate binarization, to illustrate the importance and robustness of the proposed method. As depicted in Figure 14, since a false background appears in the segmented image, the orientation correction angles are not accurate when directly derived from the LMS. With this estimated angle, an inaccurate ROI will be extracted for recognition. After detection and deletion of wrong middle points, the estimated angle is much more accurate than those without this operation, which is shown in Figure 14e. It is ascertained in Figure 14 and Table 3 that the proposed method is robust against the influences from scattering, uneven illumination, and improper image acquisition. The positive angles in Table 3 mean the image should be rotated counterclockwise, while the image should be rotated clockwise when the angle is a negative value.

In all of the acquired images in MMCBNU_6000, 3,718 images are orientation corrected. Hence, more accurate ROIs can be extracted than those without orientation correction [6].

### 4.4. Comparison of Matching Performance

The matching performance is compared in this experiment, to evaluate the effectiveness of the extracted ROIs. Three kinds of ROIs are all extracted from the correct segmented images, using the proposed segmentation method. The first method to extract ROI is using the method in [23,25,26], which means the processed images are projected to search the reference line. Image orientation correction is operated in obtaining the first ROIs. The second method is using the truncated images extracted from segmented images, to seek the reference line [6]. There is no orientation correction in the process of ROI detection. The third case, detecting ROIs from the block image truncated from processed images for reference line searching, is the proposed method.

Two conventional feature representation methods are applied to evaluate different ROIs. They are discrete wavelet transform (DWT [30]) and local binary pattern (LBP [31]). For DWT, the low frequency component after two layer wavelet decomposition using “haar” wavelet basis is applied, to give the final features. For LBP, the extracted uniform histograms from 2 × 2 nonoverlapping block images are the final features. Since the purpose of this paper is to investigate an effective and robust ROI localization method, rather than an effective feature extraction method, the parameters in these two methods are not explored more deeply. Nearest neighbor classifier with cosine distance measure is utilized for matching.

Five finger vein images from one individual are selected as the training set, while the rest five images are used as the test set. Hence, the training database and testing database are both composed of 3,000 images. Each finger is considered as an individual. To evaluate the matching performance, false accept rate (FAR) and false rejection rate (FRR) are reported. FAR and FRR will change with the variation of threshold. The equal error rate (EER) of a system is the value when FAR is equal to FRR. For the matching performance in terms of EER using different feature extraction methods, the number of genuine matching is $6,000\;(600\times C_{5}^{2})$, and the number of imposter matching is $44,925,000\;(C_{3000}^{2}-6000).$

**Figure 15.**
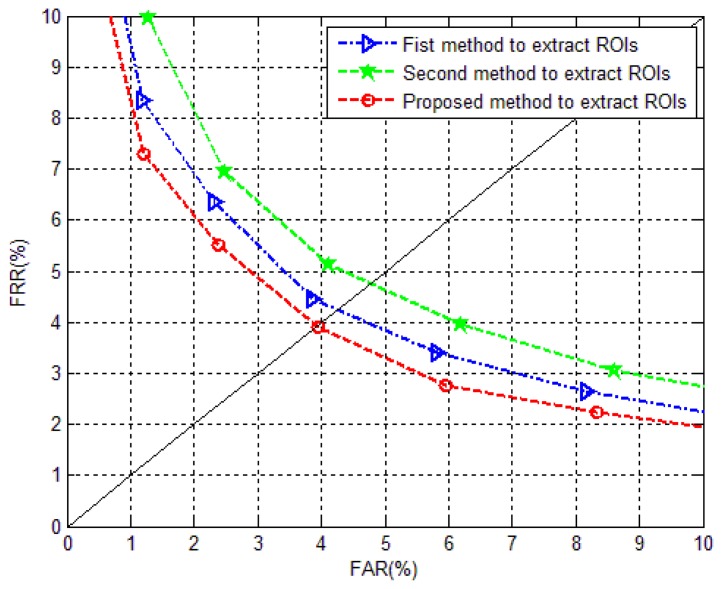
Comparison of the ROC curves obtained by using DWT for feature extraction from different kinds of ROIs.

**Figure 16.**
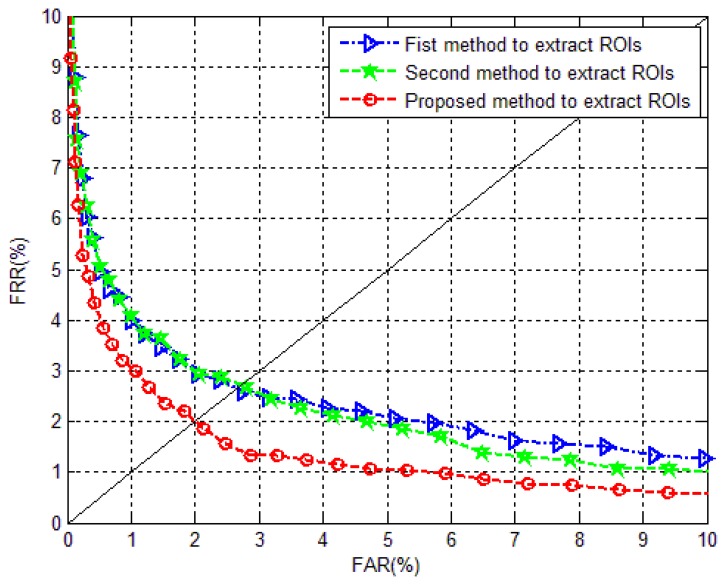
Comparison of ROC curves obtained by using LBP for feature extraction from different kinds of ROIs.

Figures 15 and 16 depict the comparative results of the ROC curves using DWT and LBP for feature extraction, respectively. It is ascertained that the ROIs extracted using the proposed method are the most effective ones, which show the smallest EERs in Table 4. The importance of image orientation correction in ROI localization is demonstrated, even the influence from image orientation is alleviated in the excogitation of a considerable finger vein imaging device. The better performance of the proposed method also demonstrates the proposed method has corrected the image orientation well. In addition, the method, of truncating the block image from the orientation corrected image for vertical projection to seek the reference line, is effective in eliminating the influences from uneven illumination, finger structure, finger pressure, *etc*.

It should be noted that the matching performance relies heavily on image enhancement method, the parameters of feature extraction method, distance metric and classifier, *etc.* The focus of this article is to investigate a robust finger vein image segmentation method for the images in MMCBNU_6000, other than an effective feature extraction method or a design of effective distance metric. The matching performance shown in Table 4 is applied to illustrate the robustness of the proposed method. The influence from image rotation (Figure 12e) and finger pressure (Figure 12f) also partly attributes the common performance shown in Table 4. Better matching performance can certainly obtained when we hammer at the researches of finger vein image enhancement, and effective feature extraction with excellent distance metric and classifier.

Table 5 shows the average processing time of different methods utilized for ROI localization. It is ascertained that all three methods can be employed in a real-time identification system. Since the ROI is extracted from the truncated image, the average processing time using the proposed method is a little less than those using the first method. With 8.7 ms consumption on image orientation correction for one image, the EER values reduce 0.75% and 0.8% for feature extraction methods using DWT and LBP, respectively.

### 4.5. Portability Analysis of the Proposed Method

In this section, we analyze the portability of the proposed method. As our best knowledge, the available finger vein databases include: the database from University of Twente (UTFVP) [32], the PKU Finger Vein Database from Peking University [33], Homologous Multi-modal Traits Database (SDUMLA-HMT) [34], and Hong Kong Polytechnic University Finger Image Database [35].

**Figure 17.**
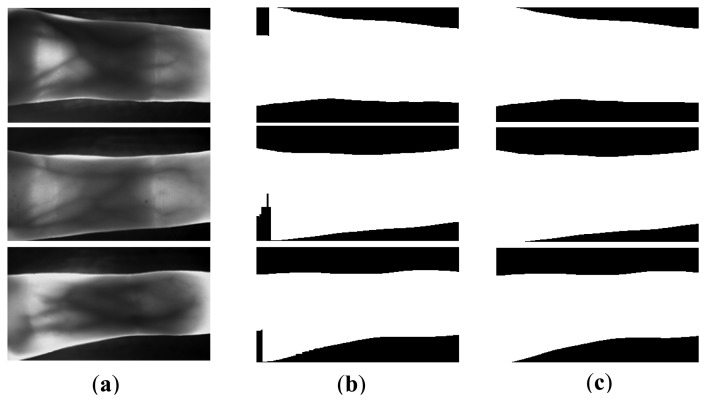
Image samples and segmented results for the images from UTFVP. (**a**) Image samples; (**b**) segmented results using mask based method proposed in [22]; and (**c**) segmented results using the proposed method.

For the images from UTFVP [32], it is illustrated from Figure 17 that the similar segmentation errors (e.g., Figure 5c3,c4) appear in the Figure 17b, when directly using the mask based method [22]. With removal of the false background using the proposed method, accurate segmentation results are achieved, as shown in Figure 17c. For 1,440 finger vein images in UTFVP, the proposed method can also achieve segmentation accuracy of 100%, which demonstrates the good portability of the proposed method. This partly attributes to the pure background of the images in UTFVP. In this experiment, the threshold *Thr* is 150 and the acquired image is reduced to half of its size.

**Figure 18.**
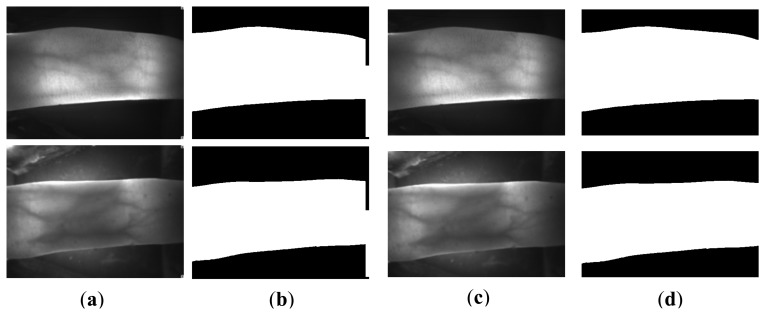
Some finger vein image samples and their corresponding segmented images using the proposed method. (**a**) images from PKU Finger Vein Database; (**b**) segmentation results using the proposed method; (**c**) images after removal of the top and bottom image tag regions; (**d**) segmentation results using the proposed method after removal of the top and bottom image tag regions.

Images included in the PKU Finger Vein Database [33] from Peking University have dark background, but the complicated inner structure of the imaging device and the image tags in the right corners of each image produce fake finger contours. Hence, inaccurate segmentation results are achieved, as shown in Figure 18b. However, when we delete the top and bottom finger parts that include the image tags (Figure 18c), accurate segmentation results are achieved by using the proposed method.

**Figure 19.**
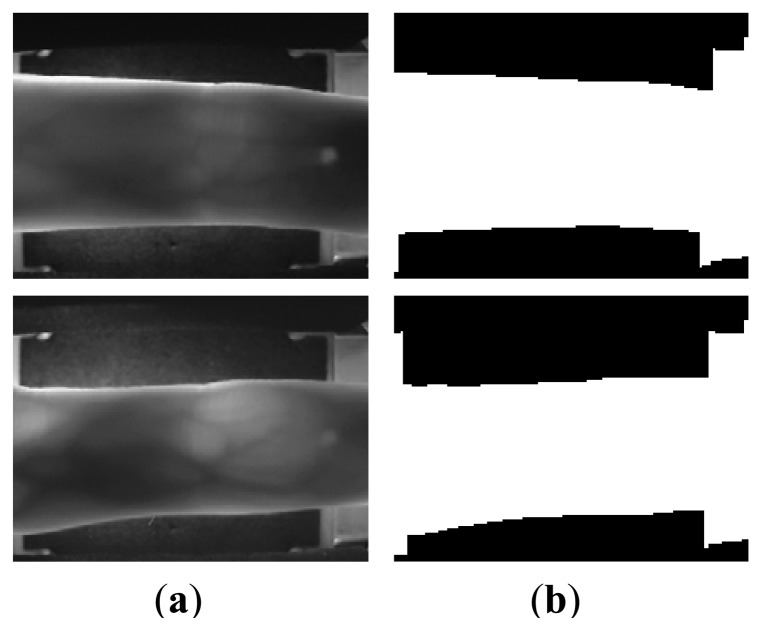
Some finger vein image samples from SDUMLA-HMT and their corresponding segmented images using the proposed method. (**a**) two finger vein images; (**b**) the corresponding segmented images using the proposed method.

As shown in Figure 19a, images included in Homologous Multi-modal Traits Database (SDUMLA-HMT) [34] also have dark backgrounds. Although the holders in the left and right sides place the fake finger contours in the region of the holders, the proposed method can also obtain accurate ROIs. As introduced in the first procedure of ROI detection, the smallest widths of the segmented images in Figure 19b can be found in the middle region. Then two edge pixels (*x*_1_, *y*_1_) and (*x*_1_, *y*_2_) can guide to do the posterior procedures, as introduced in the ROI detection part. In this case, the accurate ROIs can also be exploited using the proposed method.

**Figure 20.**
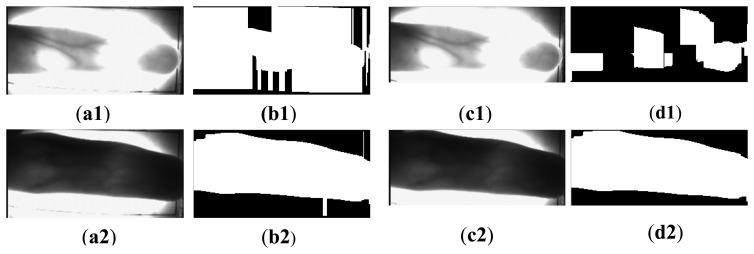
Some finger vein image samples and their corresponding segmented images using the proposed method. (**a1**–**a2**) images from Hong Kong Polytechnic University Finger Image Database [35]; (**b1**–**b2**) segmentation results using the proposed method; (**c1**–**c2**) images after removal of the top and bottom rectangle regions; (**d1**–**d2**) segmentation results using the proposed method after removal the top and bottom rectangle regions.

Images in the Hong Kong Polytechnic University Finger Image Database [35] have bright backgrounds, so we can use the proposed method for the segmentation by applying filters in reverse. The filter of Figure 3d can be used for the upper image and the filter of Figure 3c can be used for the lower image. However, the rectangle used to guide the finger brings the fake finger contour. In addition, the missing finger region, duo to the uneven illumination and the finger structure, also results in the wrong segmentation results shown in Figure 20b1,b2. To deal the influence from the black rectangle, the same method that removes the top and bottom finger parts is applied. The entire finger vein images in this database are firstly reduced to its half size, and the 4 and 14 rows of the top and bottom image parts are incised, respectively. Then the extended edge operators are performed on the processed images. By doing this, accurate segmentation results are achieved, as shown in Figure 20d2. Unfortunately, as shown in Figure 20d1, the same operation cannot solve the problem caused by the uneven illumination. The post-processing must be employed to obtain the accurate segmentation results.

Hence, with the portability analysis of the proposed method on the available databases, we can draw the conclusion that the proposed method has good portability, which can be directly used for the images from MMCBNU_6000, UTFVP [32], PKU [33] and SDUMLA-HMT [34]. Some of the parameters in this paper should be tuned according to the images in different databases. The proposed method can also be employed for the images in the Hong Kong Polytechnic University Finger Image Database [35] to get the coarse finger segmentation result. Since the images in this database have bright backgrounds, the uneven illumination can produce fake backgrounds in the finger region. Hence, additional processing must be performed to get the accurate segmentation results.

## 5. Conclusion

In this paper, we proposed a finger vein ROI localization method that has high robustness towards the influences coming from image translation, orientation, scattering, complicated background, collection posture, and uneven illumination. To eliminate or alleviate these influences, three steps, which include segmentation, orientation correction, and ROI detection, are designed for implementation. Two extended edge operators are employed in the coarse and elaborate finger region binarization. Afterwards, a single linkage clustering method is applied to discard the wrong middle points located in the false background. This ascertains the accurate estimation of the corrected orientation angle. With the estimated orientation angle, the images with false background can be filled up to get accurate segmentation results. To detect the ROI as large and effective finger region as possible, an extended ROI detection method is employed. The block image truncated from the processed image is projected to detect the reference line. This can abate the variations of uneven illumination and finger structure. Experimental results on our collected database MMCBNU_6000 have illustrated that the proposed method is robust for the aforesaid influences. In addition, the proposed method can be applied in a real-time finger vein identification system. In the future, we will devote ourselves to the investigation of a method to eliminate the influences from rotation as shown in Figure 12e and finger pressure (Figure 12f). A robust ROI localization method that has extensive applicability for finger vein images acquired by different devices also attracts our interest.

## Figures and Tables

**Table 1.** Comparison of segmentation accuracy using different methods.

**Methods**	**Otsu' Thresholding [17]**	**Mask Based Method [22]**	**Re-Binarization**	**Proposed Method**
Segmentation accuracy	86.75%	99.43%	99.92%	100%

**Table 2.** Average processing time of different segmentation methods.

**Otsu's Thresholding [17]**	**Mask Based Method [22]**	**Proposed Method**	

		**Coarse Binarization Using the Proposed Operators**	**Posterior Operation**
9 ms	8.6 ms	7.7 ms	12.3 ms

**Table 3.** Comparison of estimated orientation correction angles before and after elaborate binarization for images in Figure 14a.

**Images in Figure 14a**	**Estimated Angle before Elaborate Binarization**	**Estimated Angle after Elaborate Binarization**
First row image	−5.5709°	0.9084°
Second row image	0.7749°	−5.553°
Third row image	2.2362°	0.8465°

**Table 4.** EERs calculated using DWT and LBP for feature extraction from different kinds of ROIs.

**Feature Extraction Method**	**Equal Error Rate (EER)**		

	**First Method to Extract ROIs [23,25,26]**	**Second Method to Extract ROIs [6]**	**Proposed Method to Extract ROIs**
DWT	4.22%	4.68%	3.93%
LBP	2.63%	2.72%	1.92%

**Table 5.** Average processing time of different ROI localization methods.

**Methods to Extract ROIs**	**First Method to Extract ROIs [23,25,26]**	**Second Method to Extract ROIs [6]**	**Proposed Method to Extract ROIs**
Average processing time	22.3 ms	13.3 ms	22 ms
